# Genome-wide association analysis for β-hydroxybutyrate concentration in Milk in Holstein dairy cattle

**DOI:** 10.1186/s12863-019-0761-9

**Published:** 2019-07-16

**Authors:** S. Nayeri, F. Schenkel, A. Fleming, V. Kroezen, M. Sargolzaei, C. Baes, A. Cánovas, J. Squires, F. Miglior

**Affiliations:** 1 Centre for Genetic Improvement of Livestock, Department of Animal Biosciences, University of Guelph, Guelph, ON N1G 2W1 Canada; 2 Canadian Dairy Network, Guelph, ON N1K 1E5 Canada; 3 Select Sires Inc., Plain City, OH 43064 USA

**Keywords:** Milk BHB concentrations, MIR spectroscopy, Clinical/subclinical ketosis, Genome-wide association, Dairy cattle

## Abstract

**Background:**

Ketosis in dairy cattle has been shown to cause a high morbidity in the farm and substantial financial losses to dairy farmers. Ketosis symptoms, however, are difficult to identify, therefore, the amount of ketone bodies (mainly β-hydroxybutyric acid, BHB) is used as an indicator of subclinical ketosis in cows. It has also been shown that milk BHB concentrations have a strong correlation with ketosis in dairy cattle. Mid-infrared spectroscopy (MIR) has recently became a fast, cheap and high-throughput method for analyzing milk components. The aim of this study was to perform a genome-wide association study (GWAS) on the MIR-predicted milk BHB to identify genomic regions, genes and pathways potentially affecting subclinical ketosis in North American Holstein dairy cattle.

**Results:**

Several significant regions were identified associated with MIR-predicted milk BHB concentrations (indicator of subclinical ketosis) in the first lactation (SCK1) and second and later lactations (SCK2) in Holstein dairy cows. The strongest association was located on BTA6 for SCK1 and BTA14 on SCK2. Several SNPs on BTA6 were identified in regions and variants reported previously to be associated with susceptibility to ketosis and clinical mastitis in Jersey and Holstein dairy cattle, respectively. One highly significant SNP on BTA14 was found within the *DGAT1* gene with known functions on fat metabolism and inflammatory response in dairy cattle. A region on BTA6 and three SNPs on BTA20 were found to overlap between SCK1 and SCK2. However, a novel region on BTA20 (55–63 Mb) for SCK2 was also identified, which was not reported in previous association studies. Enrichment analysis of the list of candidate genes within the identified regions for MIR-predicted milk BHB concentrations yielded molecular functions and biological processes that may be involved in the inflammatory response and lipid metabolism in dairy cattle.

**Conclusions:**

The results of this study confirmed several SNPs and genes identified in previous studies as associated with ketosis susceptibility and immune response, and also found a novel region that can be used for further analysis to identify causal variations and key regulatory genes that affect clinical/ subclinical ketosis.

**Electronic supplementary material:**

The online version of this article (10.1186/s12863-019-0761-9) contains supplementary material, which is available to authorized users.

## Background

The increase in milk production as a result of intense genetic selection in dairy cattle has been accompanied by a higher incidence of reproductive health issues and production-related diseases, including metritis, ketosis and fatty liver [1, 2]. This can be due to the metabolic changes and challenges in high-producing dairy cows early in lactation and the failure of the cows in maintaining their internal homeostatic and homeorhetic regulations [3]. It has been reported that approximately 50% of the metabolic and infectious diseases occur during the postpartum and transition period in dairy cattle [4, 5]. Reports of eleven studies carried out in several countries have shown that the median incidence of ketosis (per cow and year) was 3.2% for the Holstein breed [6–9], which can cause a high morbidity in the farm and substantial financial losses to the dairy farmers [10, 11]. Ketosis leads to hypoglycemia and hyperketonemia, and has the highest rate of prevalence after calving that extends to the lactation peak [12, 13]. This metabolic disease can be diagnosed through clinical signs in dairy cattle, including decrease in appetite, weight loss and decrease in milk production [14]. However, these symptoms are usually difficult to detect; therefore, the amount of ketone bodies (acetone, β-hydroxybutyric acid (BHB), and acetoacetate) present in blood, milk, urine and lymph is used as good indicators of subclinical cases in cows [11, 15]. Subclinical ketosis has been defined as when the cow shows a blood BHB level of > 1.2 mmol/L [16, 17] or a non-esterified fatty acid (NEFA) concentration of > 0.4 m*M* pre-partum or > 1.0 m*M* postpartum [18]. The gold standard diagnosis of clinical and subclinical ketosis is generally BHB in blood; however, routine blood sampling is not easy to implement, expensive for the farmers [6] and stressful on the cow side [19]. Several studies showed that there may be value in measuring BHB and acetone in milk, which are closely associated with ketosis in cattle [20–22]. Mid-infrared spectroscopy (MIR) has recently become a fast, cheap and high-throughput method for analyzing chemicals in livestock and food sectors [23]. The MIR technique uses the absorption of electromagnetic radiation by a sample to determine its chemical composition and has been used to predict milk quality traits since 1960 [24]. It has been shown that the genetic correlation between ketosis and MIR predicted milk BHB in dairy cattle is strong, about 0.75 [8, 25]. Additionally, the prediction accuracy of milk BHB concentrations using fourier transform mid-infrared (FTMIR) was reported to be 71% [26].

Many studies have investigated the metabolic pathways, candidate genes and gene-networks that affect metabolic diseases (including ketosis) or metabolic energy balance in dairy cows pre and post-partum [1, 15, 27, 28]. Additionally, several genes were found to be associated with ketosis biomarkers in dairy cows [15]. However, significant genome-wide regions and genes harboring putative causative mutations have not been reported in previous studies, using the predicted milk BHB concentrations. The objective of this study was 1) to identify genome-wide regions associated with MIR predicted BHB concentrations in milk, as an indicator of sub-clinical ketosis in North American Holstein dairy cattle, and 2) to perform enrichment analysis to identify biologically significant genes and pathways associated with MIR predicted BHB concentrations in milk, as an indicator of subclinical ketosis, and their possible associations with other metabolic correlated traits.

## Results

### Association analysis

**Fig. 1:**
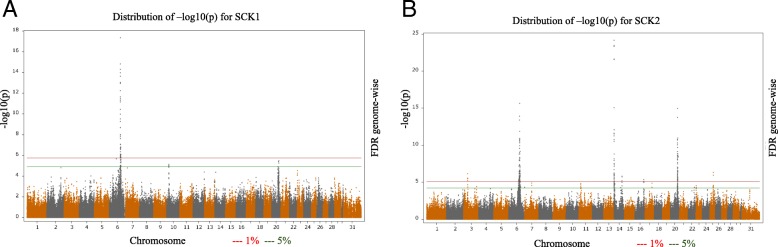
Genome-wide association analysis using single SNP regression mixed linear model for subclinical ketosis. The log_10_ of the *P*-value for association with SNPs is plotted for A. subclinical ketosis in first lactation (SCK1) and B. subclinical ketosis in later lactations (SCK2). Chromosome number is shown on the horizontal axis. The red line is the threshold for significant SNPs at 1% FDR. The green line is the threshold for significant SNPs at 5% FDR

**Fig. 2:**
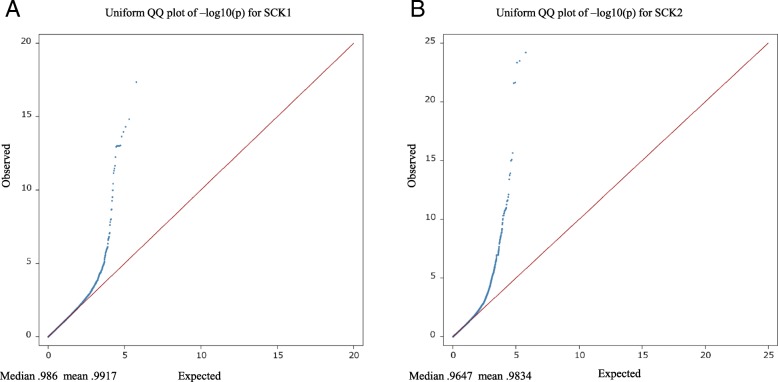
Quantile-quantile (Q-Q) of *P*-values of SNPs from single SNP regression mixed linear model for subclinical ketosis. The blue dots represent the –log_10_(P-values) of the observed test statistics for no association for **a**. subclinical ketosis in first lactation (SCK1) and **b**. subclinical ketosis in later lactations (SCK2). The red line denotes the expected pattern under the null hypothesis. Deviations between the red line and blue dots indicate how the observed probability of the test statistics deviate from the expected

Association analyses using a single SNP regression mixed linear model identified strong associations for the MIR predicted milk BHB from the first (SCK1) and later lactations (SCK2). The –log_10_ (*P*-value) of the tested SNPs from the GWAS analyses are shown as Manhattan plots for SCK1 (Fig. 1a) and SCK2 (Fig. 1b). The number of significant SNPs identified at a 5% genome-wise FDR varied from 71 for SCK1 to 369 SNPs for SCK2 (Additional file 1). The Quantile-Quantile (Q-Q) plots and genomic inflation statistics (lambda) of the two GWAS analyses are shown in Fig. 2a and Fig. 2b for SCK1 and SCK2, respectively. The plots showed no evidence of inflation of model-based statistics with a λ_median_ = 0.986 for SCK1 and λ_median_ = 0.964 for SCK2 traits.

### Annotations and network-based analysis

**Fig. 3:**
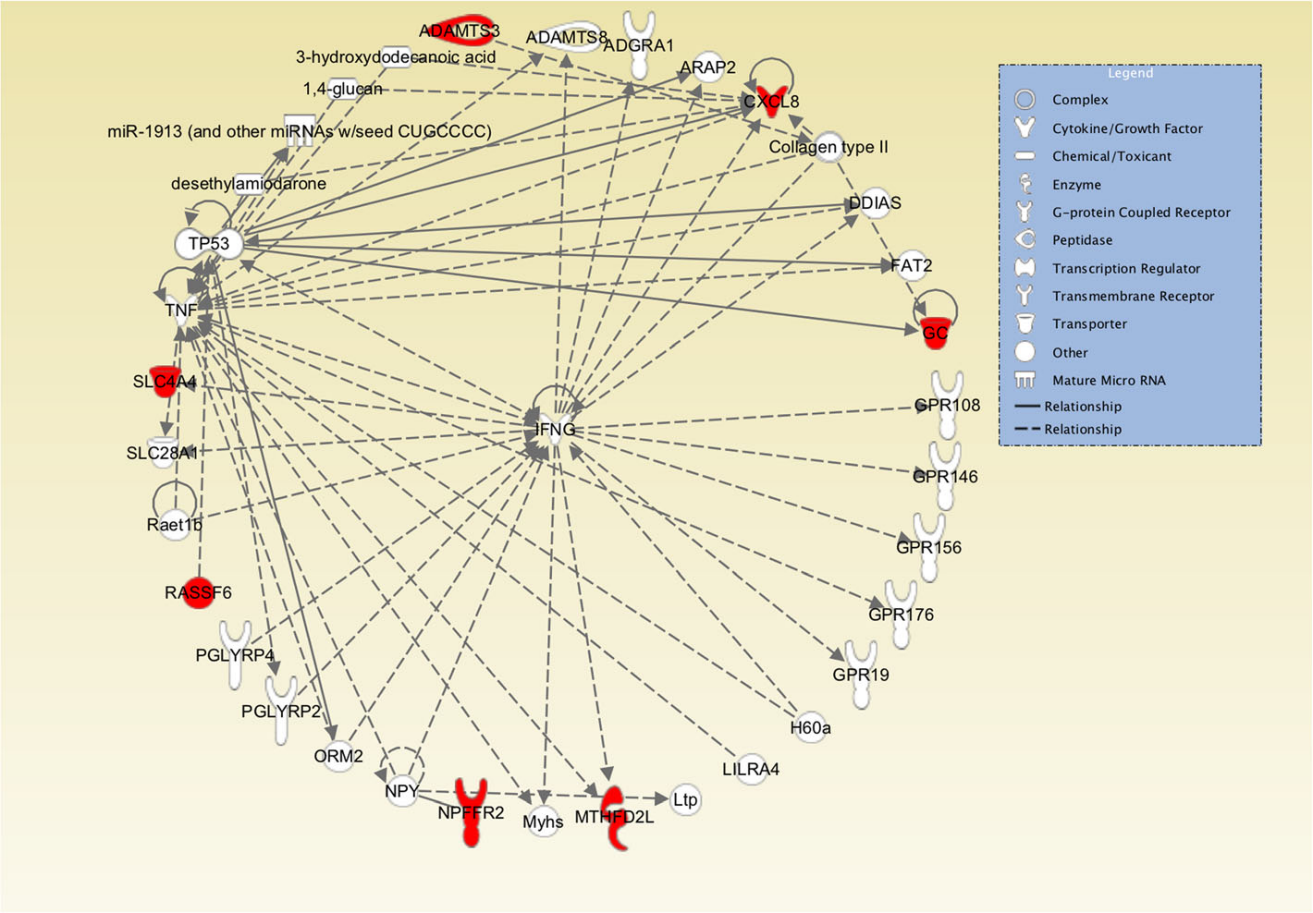
Network of interactions between GWAS candidate genes for subclinical ketosis in first lactation (SCK1) using ingenuity pathway analysis (IPA). The network shows direct and indirect interactions between the candidate genes enriched for significantly associated SNPs (FDR < 5%) with two gene hubs tumor necrosis factor (TNF) and interferon gamma (IFNG) as two important immune system regulators. The red label indicates the genes that were enriched for significantly associated SNPs. All the relationships were obtained using information contained in the IPA repository

**Fig. 4:**
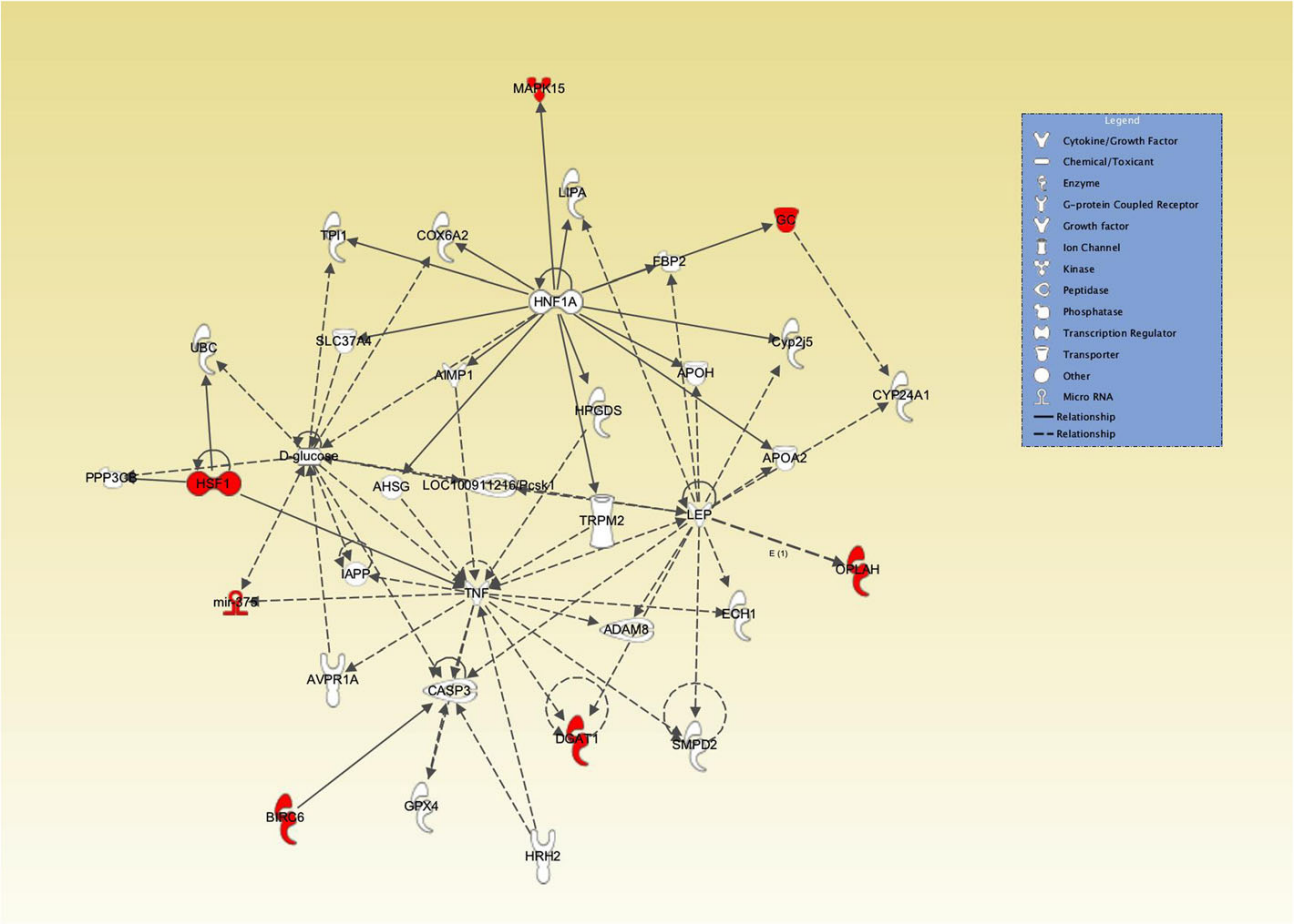
Network of interactions between GWAS candidate genes for subclinical ketosis in later lactations (SCK2) using ingenuity pathway analysis (IPA). The network shows direct and indirect interactions between the candidate genes enriched for significantly associated SNPs (FDR < 5%) with two gene hubs tumor necrosis factor (*TNF*) and leptin (*LEP*) as two important genes involved in immune system and lipid metabolism regulators. The red label indicates the genes that were enriched for significantly associated SNPs. All the relationships were obtained using information contained in the IPA repository

Annotation of the genes to the corresponding significant SNPs from the GWAS resulted in identification of nine unique genes for SCK1 (Table 1) and 86 unique genes for SCK2 (Table 2). Gene-network analysis was performed using the IPA tool. Using two gene lists, the IPA analysis produced two putative networks for SCK1 and seven networks for the SCK2. The top two putative gene-networks for the SCK1 were associated with disease and functions involved in hematological diseases, organismal injuries and abnormalities, and infectious diseases. For the SCK2, several of the networks resulting from IPA were related to general molecular and cellular processes, including cell death and survival, molecular transport and cellular development. Most of these networks were also involved in more specific pathways such as developmental disorders, embryonic development, endocrine disorders, and metabolic diseases. The most informative networks and molecular interactions, in the context of this study, were the networks, interacting molecules and genes grouped around interferon gamma (*IFNG*), and tumor necrosis factor (*TNF*) with IPA score of 20 for SCK1 (Fig. 3), and leptin (*LEP*) gene with IPA score 12 for SCK2 (Fig. 4).

**Table 1:** List of candidate genes and corresponding significant identified SNPs within or very close (±100 kb) to the SNPs for subclinical ketosis in first lactation (SCK1)

SNP_name	Rs_snp_name	chromosome	position	Ensembl_gene _id	Entrez_gene_id
BovineHD0600024204; BovineHD0600024173; BovineHD0600024179; BovineHD0600024201	rs110246305; rs134989847; rs109811572; rs109901151	6	88498400; 88433671; 88441084; 88494442	ENSBTAG00000002348	SLC4A4
BovineHD0600025613	rs109510145	6	92951049	ENSBTAG00000004921	FAM47E
Hapmap44452-BTA-22099	rs41606699	6	89399736	ENSBTAG00000006507	ADAMTS3
BovineHD0600024406	rs137844449	6	89050323	ENSBTAG00000009070	NPFFR2
BovineHD0600026322	rs110306794	6	94884203	ENSBTAG00000010716	FRAS1
BovineHD0600024752; BovineHD0600024746	rs110185652; rs135247183	6	90382995; 90371709	ENSBTAG00000011935	RASSF6
BovineHD0600024291; BovineHD0600024289; BovineHD0600024294;BovineHD0600024290	rs109547247; rs110310151; rs137023501; rs43338561	6	88732184; 88728581; 88735599; 88730717	ENSBTAG00000013718	GC
BovineHD0600024791	rs109793149	6	90557327	ENSBTAG00000019716	CXCL8
BovineHD0600024925; BovineHD0600024923	rs110789319; rs133534783	6	90982259; 90977968	ENSBTAG00000043960	MTHFD2L
BovineHD0600024204; BovineHD0600024173; BovineHD0600024179; BovineHD0600024201	rs110246305; rs134989847; rs109811572; rs109901151	6	88498400; 88433671; 88441084; 88494442	ENSBTAG00000002348	SLC4A4
BovineHD0600025613	rs109510145	6	92951049	ENSBTAG00000004921	FAM47E
Hapmap44452-BTA-22099	rs41606699	6	89399736	ENSBTAG00000006507	ADAMTS3
BovineHD0600024406	rs137844449	6	89050323	ENSBTAG00000009070	NPFFR2
BovineHD0600026322	rs110306794	6	94884203	ENSBTAG00000010716	FRAS1
BovineHD0600024752; BovineHD0600024746	rs110185652; rs135247183	6	90382995; 90371709	ENSBTAG00000011935	RASSF6
BovineHD0600024291; BovineHD0600024289; BovineHD0600024294;BovineHD0600024290	rs109547247; rs110310151; rs137023501; rs43338561	6	88732184; 88728581; 88735599; 88730717	ENSBTAG00000013718	GC
BovineHD0600024791	rs109793149	6	90557327	ENSBTAG00000019716	CXCL8
BovineHD0600024925; BovineHD0600024923	rs110789319; rs133534783	6	90982259; 90977968	ENSBTAG00000043960	MTHFD2L

**Table 2:** List of candidate genes and corresponding significant identified SNPs within or very close (±100 kb) to the SNPs for subclinical ketosis in second and later lactations (SCK2)

SNP_name	rs_snp_name	chromosome	position	Ensembl_gene _id	Entrez_gene_id
BovineHD0300010696; BovineHD0300010697	rs109078663; rs110789481	3	34314659; 34316156	ENSBTAG00000001136	KIAA1324
BovineHD0300010081	rs42784745	3	32149527	ENSBTAG00000023535	NA
BovineHD0300010059	rs43333803	3	32082026	ENSBTAG00000034841	NA
Hapmap60224-rs29001782	rs29001782	6	85178107	ENSBTAG00000000438	GNRHR
BovineHD0600024201; BovineHD0600024204; Hapmap54015-rs29022799;	rs109901151; rs110246305; rs29022799; rs43473311	6	88494442; 88498400; 88421804; 88212890	ENSBTAG00000002348	SLC4A4
Hapmap50857-BTA-107920	rs41610991	6	86819633	ENSBTAG00000004040	UGT2A1
BovineHD0600025086	rs42606224	6	91447170	ENSBTAG00000004237	BTC
BovineHD0600025613; BovineHD0600025593	rs109510145; rs137505966	6	92951049; 92919992	ENSBTAG00000004921	FAM47E
BovineHD0600025431; BovineHD0600025432; BovineHD0600025421	rs133065719; rs134889097; rs43476314	6	92584159; 92585321; 92567521	ENSBTAG00000007639	SDAD1
BovineHD4100005289; BovineHD4100005290	rs110786992; rs110899052	6	87136725; 87145250	ENSBTAG00000007695	CSN1S1
BovineHD0600024406	rs137844449	6	89050323	ENSBTAG00000009070	NPFFR2
BovineHD0600022837; BovineHD0600022838; BovineHD0600022835; BovineHD0600022833; BovineHD0600022839; BovineHD0600022830; BovineHD0600022831; BovineHD0600022834	rs132864694; rs134101925; rs134383135; rs135577932; rs135693518; rs136048407; rs136499310; rs136560941	6	82920849; 82925435; 82912559; 82905087; 82929947; 82896236; 82898792; 82907694	ENSBTAG00000009438	EPHA5
BovineHD0600024752	rs110185652	6	90382995	ENSBTAG00000011935	RASSF6
BovineHD0600023861	rs135052123	6	87039570	ENSBTAG00000011952	SULT1E1
ARS-BFGL-NGS-112872	rs110707460	6	88069548	ENSBTAG00000012397	DCK
BovineHD0600024291; BovineHD0600024289; BovineHD0600024288; BovineHD0600024294; BovineHD0600021907; Hapmap52479-rs29018853	rs109547247; rs110310151; rs133300430; rs137023501; rs132666893; rs29018853	6	88732184 88728581; 88724389; 88735599; 79273130; 79203343	ENSBTAG00000013718	GC
BovineHD0600023797	rs135797682	6	86686282	ENSBTAG00000015047	LOC781988
BovineHD0600023599; BovineHD0600023609	rs135335675; rs136617775	6	85838311; 85861013	ENSBTAG00000015572	YTHDC1
BovineHD0600024791	rs109793149	6	90557327	ENSBTAG00000019716	CXCL8
Hapmap43767-BTA-113302	rs41618641	6	85646902	ENSBTAG00000038520	NA
Hapmap60224-rs29001782	rs29001782	6	85178107	ENSBTAG00000038648	LOC100140490
BovineHD0600023676	rs136388495	6	86188921	ENSBTAG00000039991	UGT2B10
BovineHD0600023659	rs136518898	6	86122072	ENSBTAG00000040337	NA
BovineHD0600024923; BovineHD0600024884	rs133534783; rs133586557	6	90977968; 90863255	ENSBTAG00000043960	MTHFD2L
BovineHD0600024601	rs135618950	6	89677070	ENSBTAG00000047335	NA
BovineHD0600023628	rs110240917	6	85933714	ENSBTAG00000048013	LOC100138004
BovineHD1100004827; BovineHD1100004831	rs110571223; rs43672311	11	15050414; 15081731	ENSBTAG00000027932	BIRC6
BovineHD1100004868; BovineHD1100004913	rs110243275; rs42050566	11	15212371; 15369971	ENSBTAG00000033010	TTC27
BovineHD1400000443; BovineHD1400000446	rs133518905; rs136122630	14	2758369; 2763621	ENSBTAG00000000158	LY6K
BovineHD1400000275	rs133271979	14	2019390	ENSBTAG00000000312	GRINA
Hapmap52798-ss46526455	rs41256919	14	1923292	ENSBTAG00000000658	WDR97
Hapmap30383-BTC-005848	rs109752439	14	1489496	ENSBTAG00000000879	C14H8orf33
Hapmap30374-BTC-002159	rs109529219	14	2468020	ENSBTAG00000002104	RHPN1
Hapmap30086-BTC-002066; BovineHD1400000389	rs110199901; rs110382236	14	2524432; 2518104	ENSBTAG00000003606	NA
BovineHD1400018136; BovineHD1400018139	rs135447252; rs41632852	14	64993498; 64996863	ENSBTAG00000004518	GRHL2
ARS-BFGL-NGS-3122	rs109476905	14	2721633	ENSBTAG00000004595	GML
BovineHD1400000434	rs110295964	14	2707911	ENSBTAG00000004596	LOC787628
ARS-BFGL-NGS-57820	rs109146371	14	1651311	ENSBTAG00000004761	FOXH1
BovineHD1400000187;	rs136580003	14	1585385	ENSBTAG00000007186	ARHGAP39
ARS-BFGL-NGS-94706	rs17870736	14	1696470	ENSBTAG00000007749	TONSL
BovineHD1400000305	rs137757978	14	2164419	ENSBTAG00000008079	NRBP2
BovineHD4100010523; BovineHD4100010524; UA-IFASA-8997	rs110288957; rs110502044; rs41602530	14	2198215; 2201929; 2194228	ENSBTAG00000008421	SCRIB
ARS-BFGL-NGS-107379; BovineHD1400000288	rs109350371; rs135270011	14	2054457; 2084067	ENSBTAG00000011922	LOC786966
BovineHD1400000400; BovineHD4100010548	rs109221516; rs110651119	14	2568452; 2566015	ENSBTAG00000011939	LY6H
Hapmap52798-ss46526455	rs41256919	14	1923292	ENSBTAG00000012235	SHARPIN
Hapmap52798-ss46526455	rs41256919	14	1923292	ENSBTAG00000012242	MAF1
Hapmap30383-BTC-005848	rs109752439	14	1489496	ENSBTAG00000012353	ZNF34
BovineHD1400000239; BovineHD1400000246	rs133299034; rs137787931	14	1855090; 1880378	ENSBTAG00000014458	MROH1
BovineHD1400000262	rs135549651	14	1967325	ENSBTAG00000015040	SMPD5
BovineHD1400000457	rs135781491	14	2833552	ENSBTAG00000016209	SLURP1
BovineHD1400000457	rs135781491	14	2833552	ENSBTAG00000016210	LYPD2
BovineHD1400000262	rs135549651	14	1967325	ENSBTAG00000017281	OPLAH
BovineHD1400018541	rs136940827	14	66276813	ENSBTAG00000017833	RNF19A
BTA-34956-no-rs	rs41630614	14	1514056	ENSBTAG00000018456	ZNF7
BovineHD1400018582	rs134747221	14	66468090	ENSBTAG00000019793	RGS22
ARS-BFGL-NGS-4939	rs109421300	14	1801116	ENSBTAG00000020751	HSF1
BovineHD4100010534	rs110706284	14	2398876	ENSBTAG00000021472	ZC3H3
ARS-BFGL-NGS-94706	rs17870736	14	1696470	ENSBTAG00000026320	NA
BovineHD1400000474; BovineHD1400000480; BovineHD1400000473; BovineHD1400000482	rs108968192; rs136634846; rs137202573; rs137438227	14	2916658; 2936478; 2915391; 2940147	ENSBTAG00000026340	NA
BovineHD1400000262; BovineHD1400000271; UA-IFASA-6878	rs135549651; rs136792973; rs41629750	14	1967325; 2002126; 2002873	ENSBTAG00000026350	NA
ARS-BFGL-NGS-4939	rs109421300	14	1801116	ENSBTAG00000026356	DGAT1
ARS-BFGL-NGS-34135	rs109968515	14	1675278	ENSBTAG00000035254	CYHR1
BovineHD1400000305	rs137757978	14	2164419	ENSBTAG00000037493	PUF60
BovineHD1400000324	rs137181538	14	2257386	ENSBTAG00000040360	CCDC166
BovineHD1400000246	rs137787931	14	1880378	ENSBTAG00000044406	MIR1839
BovineHD1400000300; BovineHD4100010521	rs109342545; rs110053839	14	2147133; 2135276	ENSBTAG00000045727	NA
BovineHD1400000706	rs135132676	14	3583221	ENSBTAG00000046467	PTP4A3
BTA-35941-no-rs	rs41627764	14	2276443	ENSBTAG00000046866	NA
BovineHD1400024339	rs136939758	14	1308359	ENSBTAG00000046932	NA
BovineHD1400000462	rs132789965	14	2857000	ENSBTAG00000047022	PSCA
BovineHD1700015848	rs137100268	17	55864167	ENSBTAG00000004457	ORAI1
BovineHD2000016332	rs109823133	20	58638275	ENSBTAG00000000672	OTULINL
ARS-BFGL-NGS-84494	rs109643490	20	57301571	ENSBTAG00000003219	FBXL7
BovineHD2000016490; BovineHD2000016482; BovineHD2000016480; BovineHD2000016463; ARS-BFGL-NGS-71622; BovineHD2000016473; BovineHD2000016468; BovineHD2000016401; BovineHD2000016466; BovineHD2000016477; BovineHD2000016465; BovineHD2000016474; BovineHD2000016471; BovineHD2000016486; BovineHD2000016464; BovineHD2000016485; BovineHD2000016476; BovineHD2000016470; BovineHD2000016479; BovineHD2000016445; BovineHD2000016487; BovineHD2000016478; BovineHD2000016491; BovineHD2000016475; BovineHD2000016469; BovineHD2000016481; BovineHD2000016467; BovineHD2000016394; BovineHD2000016484; BovineHD2000016392; BovineHD2000016472; BovineHD2000016364;	rs108957596; rs108964368; rs109059391; rs109148196; rs109243453; rs109671324; rs109939825; rs110078451; rs110093810; rs110410028; rs110554011; rs110608216; rs110681006; rs110881846; rs132765887; rs133138163; rs133624494; rs133732006; rs133761028; rs134168996; rs135516192; rs135821345; rs135888983; rs136263201; rs136340192; rs136561486; rs136576574; rs136944246; rs137465525; rs137486152; rs137528125; rs41950612	20	58947745; 58932752; 58929832; 58904206; 58928800; 58922307; 58915583; 58796750; 58911097; 58925687; 58908876; 58923230; 58919595; 58936512; 58906478; 58935497; 58925004; 58917974; 58927712; 58878031; 58938683; 58926579; 58948689; 58924069; 58916701; 58931087; 58914355; 58771588; 58934789; 58762885; 58920803; 58709513	ENSBTAG00000005514	TRIO
BovineHD2000016248; ARS-BFGL-NGS-111931; BovineHD2000016258; BovineHD2000016246; BovineHD2000016226; BovineHD2000016190; BovineHD2000016253; BovineHD2000016252; BovineHD2000016203; BovineHD2000016239; BovineHD2000016237; BovineHD2000016240; BovineHD2000016232; BovineHD2000016238; BovineHD2000016241; BovineHD2000016236; BovineHD4100014800; BovineHD2000016206; BovineHD2000016208; BovineHD2000016210; BovineHD2000016227; BovineHD2000016216; BovineHD2000016217; BovineHD2000016218	rs108968188; rs109102596; rs109167035; rs109439555; rs110470671; rs110589026; rs110630125; rs110862310; rs110983331; rs133021030; rs133648068; rs134090885; rs134817611; rs135781363; rs135920407; rs137840204; rs41642545; rs41951107; rs41951130; rs41951335; rs42640155; rs42640157; rs42640164; rs42640170	20	58493410; 58405641; 58522200; 58491204; 58445208; 58374946; 58501904; 58500982; 58417934; 58465508; 58462053; 58468597; 58455297; 58463540; 58470255; 58459086; 58392839; 58421319; 58425019; 58426752; 58445701; 58433919; 58435724; 58436944	ENSBTAG00000013391	ANKH
BovineHD2000015474; BovineHD2000015529	rs137602919; rs42809077	20	56327023; 56449013	ENSBTAG00000020126	MYO10
BovineHD2000016591; BovineHD2000016689; UA-IFASA-2314; BovineHD2000016639	rs110554444; rs134140692; rs29012816; rs41953112	20	59283222; 59484278; 59514189; 59416887	ENSBTAG00000021972	DNAH5
BovineHD2000016248; BovineHD2000016246	rs108968188; rs109439555	20	58493410; 58491204	ENSBTAG00000045869	NA
BovineHD2500007120	rs42062457	25	25174707	ENSBTAG00000001602	IL4R
BovineHD2500007345	rs135129780	25	25895888	ENSBTAG00000010309	XPO6
BovineHD2500007818	rs109700166	25	27974664	ENSBTAG00000013081	PSPH
BovineHD2500007447	rs135166725	25	26358012	ENSBTAG00000016883	NA
BovineHD2500007435; BovineHD2500007432	rs42071217; rs42072596	25	26317395; 25	ENSBTAG00000018000	CLN3
BovineHD2500007435	rs42071217	25	26317395	ENSBTAG00000018011	APOBR
BovineHD2500007435	rs42071217	25	26317395	ENSBTAG00000018015	IL27

**Fig. 5:**
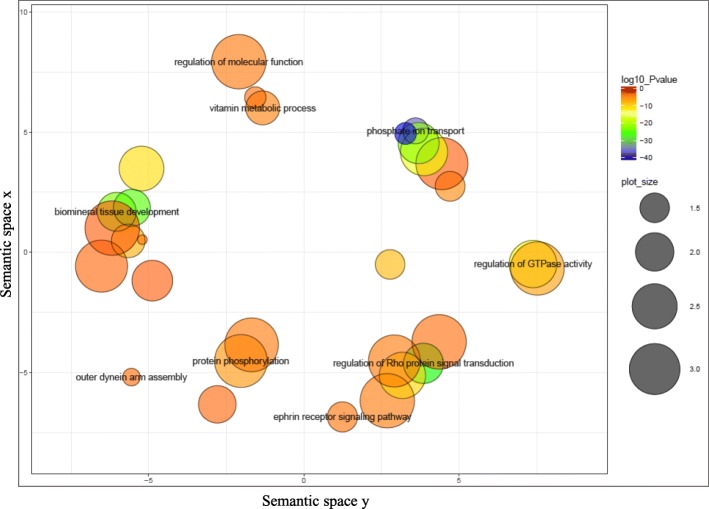
Gene Ontology scatterplot constructed using REVIGO in R [37] for all the GO terms and genes within the identified significant regions for subclinical ketosis. Settings used for REVIGO program were as follow: database: *Bos taurus*, semantic similarity: 0.5 (small), semantic similarity measure: SimRel. Colors indicate the *P*-value of the enriched GO terms provided by the authors. The size of each bubble shows the GO terms with more significant *P*-values. The size of the bubbles indicates the frequency of the GO terms

The candidate SNP enrichment analysis resulted in enrichment of seven biologically significant GO terms for SCK1 and 91 significant GO terms for SCK2 (Additional file 2), potentially involved in pathways affecting subclinical ketosis. The semantic similarities among the enriched GO terms (FDR < 1%) using REVIGO is shown in Fig. 5.

## Discussion

### Subclinical ketosis in first lactation (SCK1)

Most of the identified significant SNPs (FDR < 1%) for SCK1 in this study were located on *Bos taurus autosome* (BTA) 6 (Fig. 1a, Additional file 1). These SNPs were located at around 88.4~94.8 Mb and this region has not been reported to be significantly associated with ketosis or subclinical ketosis in previous investigations. The long length of regions identified here might be associated with the imputation process. As a result of imputing, missing SNPs and more SNPs and haplotypes (in possible linkage-disequilibrium (LD) with the casual mutations) are added to the dataset. In addition, a Holstein cattle sample commonly has close relatives, who share long haplotypes. Having long haplotypes in LD with the causal mutation might be a reason for the long QTL region identified by the GWAS.

One single significant SNP (FDR < 5%) on BTA6 (BOVINEHD0600015345: rs109930261) was located at 56 Mb (Additional file 1). The position of this SNP is close to the position of several SNPs reported in a recent study on ketosis susceptibility in US Jersey cattle [29]. Parker et al. (2018) reported that the largest proportion of the variance for the ketosis was associated with a region on chromosome 6 at 56.1 Mb [29]. This region was shown to be associated with the genes and functions that affect basal glucose uptake and regulation of glucose transporter *GLUT1* and lipid droplet formation [29, 30]. Association of this region on chromosome 6 with milk metabolites, glucose and glutamate, and left-sided displaced abomasum has been also reported in Danish [31] and German [32] Holstein cattle, respectively. Another highly significant (FDR < 1%) SNP identified on BTA6 for SCK1 (BovineHD0600024791: rs109793149) at around 90.5 Mb is located in the gene C-X-C motif chemokine ligand 8 (*CXCL8*). The protein encoded by this gene is a major mediator of the inflammatory response, secreted primary by neutrophils and acts at the site of infection (Gene ID: 3576). A study of the effect of the ketone body BHB on innate defense capability of the bovine mammary epithelial cells showed that expression of this gene (*CXCL8*) was significantly induced 30 h post infection with *E. coli* bacteria [28]. The result of this study is in agreement with previous researches reporting important interactions between metabolic disease and immune response in dairy cow [33, 34]. The association of several genes with key functions in immune response has also been reported in a previous study [29]. Several other candidate genes, including *SLC4A4*, *GC*, *NPFFR2*, *ADAMTS3*, *RASSF6* and *MTHFD2L*, were identified in this study on BTA6, located within the region 88~90 Mb. The *SLC4A4* gene (Solute carrier Family 4, Member 4) is a protein coding gene that encodes a sodium bicarbonate cotransporter (Na^+^/HCO^−^ _3_ cotransporter) involved in the regulation of bicarbonate secretion, absorption and intracellular pH (ID: 8671). The importance of this gene along with other ketogenic genes in various metabolic pathways as well as intracellular pH control in the rumen epithelium tissue was previously reported in neonatal Holstein calves [35]. Another region for milk BHB concentration harbors a functional candidate gene named *GC* gene. A fine mapping study of a QTL on bovine chromosome 6 using imputed full sequence data suggested that the *GC* gene has a key role in clinical mastitis and milk production in dairy cattle [36]. The *GC* gene encodes vitamin D-binding protein (DBP) which is the main carrier of vitamin D3. In both human and bovine, during infection, monocytes and macrophages express and produce and enzyme that converts vitamin D to its biologically active form (25(OH)D3), which has an important role in the immune system and host defense of the animal [36–38]. Moreover, concentrations of 25(OH)D3 are reported to be associated with pregnancy cycle and lactation stage [39]. The *NPFFR2* gene (neuropeptide FF receptor 2) along with *GC* and *ADAMTS3 (ADAM metallopeptidase with thrombospondin type 1 motif* 3) genes were reported previously to be located within a QTL region affecting clinical mastitis in Norwegian Red dairy cattle [36]. Several regions and genes identified in this study associated with BHB in milk were previously reported to be associated with inflammatory response. In this regard, Drackley [40] and Contreras and Sordillo [41] have proposed that inflammation is the missing link in the pathology of metabolic disorders in transition cows. Intense lipid metabolism, during the transition period, results in significant release of non-esterified fatty acids (NEFA) into the blood stream which can directly activate NF-ĸB signaling pathways [41, 42]. The NF-ĸB can induce the expression of several cytokines, chemokines and their receptors and subsequently enhance the cellular inflammatory response [42]. The metabolic effects of the acute systematic inflammation in transition cow results in mobilization of more adipose tissue, liver glycogen breakdown [43] and accumulation of the triglyceride in the liver [42]. All of these events are associated with ketosis and fatty liver in dairy cattle [44]. Taking all these results together, the authors of this study speculate that this region on BTA6 and the associated candidate genes might regulate pathways and events related to energy metabolism, inflammatory response function and cellular crosstalk in subclinical ketosis.

The GWAS results also showed three significant SNPs (BOVINEHD1000005959: rs110237995; BOVINEHD1000005943: rs110139536 and BOVINEHD1000005952: rs135236614, FDR < 5%) located on BTA10 at around 17 Mb (Additional file 1). Parker et al. (2018) reported a region on chromosome 10 associated with ketosis susceptibility; however, the position of this region was different from what was found in the current study. Three significant SNPs (FDR < 5%, Additional file 1) on chromosome 20 spanning around 55.3–57.4 Mb were also identified significant in this study. Two of these SNPs in this region (BOVINEHD2000015856: rs133951346; BOVINEHD2000015861: rs135815835) were located within the intronic region of gene F-box and leucine rich repeat protein 7 (*FBXL7*). The *FBXL7* gene includes one of the four subunits of the E3 ubiquitin protein ligases, which play a role in phosphorylation-dependent ubiquitination of proteins (Gene ID: 23194). The ubiquitin system plays a pivotal role in the regulation of immune response and controls the basic aspect of the immune system, including lymphocyte development, differentiation and activation [45, 46]. The *FBXL7* gene has also been reported to be located within a region that explains the highest genetic variance associated with clinical mastitis in first lactating US Holstein dairy cattle [47]. Therefore, this gene might be associated with inflammation factors that affect both clinical mastitis and clinical ketosis during first lactation in dairy cattle.

The IPA network enrichment analysis of the genes identified for SCK1 and their direct and indirect interactions with other molecules and genes are shown in Fig. 3. This network shows the association of most of the identified genes for SCK1 with two hub genes, *IFNG* and *TNF*. The *IFNG* gene encodes a soluble cytokine that is a member of type II interferon class and has important immune-regulatory functions in both innate and adaptive immune systems (Gene ID: 3458). In addition, cows with metritis or clinical endometritis show higher serum concentrations of leptin, adiponectin, IL-1β, IL-6and TNF-α [48]. In transition dairy cows, once mobilized NEFA reach the liver, the TNF-α decreases liver glucose production [43] and increase the triglyceride accumulation which can promote metabolic disorders [42]. Kasimanickam et al. [48] also reported that an increase in anti- and pro-inflammatory cytokines early in lactation is associated with uterine postpartum pro-inflammatory conditions, increase in adipose tissue metabolism and loss of body condition score; these last two factors are key biological features that affect ketosis and subclinical ketosis in dairy cattle [28, 29, 48].

Candidate SNP enrichment analysis for SCK1 has resulted in enrichment of seven significant GO terms (FDR ≤ 1%), introducing 71 SNPs as candidate SNPs (Additional file 2). From this significant GO terms, several terms including GO:0051180 (vitamin transport), GO:0051183 (vitamin transmembrane transport activity) and GO:0042359 (vitamin D metabolic process) were related to vitamin transport within and between cells and chemical reactions and pathways involving vitamin D (Additional file 2). Vitamin D has been reported to have regulatory effects on adipose tissue, lipid storage and calcium metabolism in humans and it has been shown that vitamin D receptor (VDR) is expressed in adipose tissue [49]. It has also been reported that plasma 25-hydroxy-vitamin D3(25OHD3) is inversely related to metabolic events and obesity complications, including hyperglycemia, hypertension, insulin resistance and dyslipidemia in humans [50].

### Subclinical ketosis in second and later lactations (SCK2)

A total number of 369 SNPs (FDR < 5%) were identified significant for SCK2 in this study. These SNPs were mainly located on BTA6, 14 and 20 (Fig. 1b). The list of all significant SNPs and chromosomes are given in the Additional file 1.

The presence of these significant regions on chromosomes (6, 14 and 20) for SCK2 was reported in a previous short paper by Nayeri et al. [51] where the largest peak was located on BTA14 (Fig. 1b). In the following, the position of significant SNPs, their functional associations and other identified significant regions will be further discussed. One highly significant SNP on BTA14 (ARS-BFGL-NGS-4939: rs109421300, FDR < 1%) was shown to be an intronic SNP within *DGAT1* gene (Additional file 1). Association of the *DGAT1* gene with milk fat content [52, 53] and several production traits has been reported in several previous studies [53–55]. However, there were no previous indications of association of the identified SNP in this region with ketosis in previous studies. The enzyme produced by the *DGAT1* gene (acyl-CoA: diacylglycerol acyltransferase) catalyzes the last step of triacyglyceride (TAG) synthesis in the liver and can significantly influence the energy metabolism in dairy cows [56]. It has been shown that excessive accumulation of TAG in liver and oxidative stress as a result of altered lipid metabolism early in lactation can increase the risk of metabolic disease including fatty liver and ketosis in dairy cattle [56]. Estimates of genetic correlations between 305-d milk yield and ketosis were found to be mostly unfavorable, with estimates ranging from 0.00 to 0.77 [57–59]. Therefore, one would expected QTL/genes with pleiotropic effects on both ketosis and milk yield would exist. This seems to be observed in this study, but the mechanism of the pleiotropic effects might involve intricate indirect pathways. These findings may also support the IPA enrichment analysis results, in which the DGAT1 enzyme was shown to be interacting indirectly with *LEP* and *TNF* genes (Fig. 4). Leptin is a hormone produced in the adipose tissue and its function is mainly to maintain glucose homeostasis and regulate appetite and energy metabolism in dairy cattle [60]. The *TNF* gene encodes a pro-inflammatory cytokine that is mainly secreted by macrophages; this cytokine, as explained previously, is involved in regulation of different biological processes including cell differentiation, proliferation, apoptosis and lipid metabolism and has been implicated in different autoimmune and metabolic-associated diseases including insulin resistance (Gene ID: 7124). Investigations on human and various animal models suggest that fatty acids can affect the host inflammatory responses in several ways [61]. One way is through the altered lipid metabolism, which often results in the increased levels of NEFA in the blood. Fatty acids are key resources of energy metabolism and are oxidized to produce Acyl-CoA and ATP. When there is a nutrient surplus, adipose tissue stores a large quantity of fatty acids in the form of TAGs [3]. This stored fatty acids can be then mobilized by lipolysis when there is an energy deficit, including early in lactation in dairy cattle [62, 63], which leads to increased NEFA in the blood for uptake by various tissues [64]. However, other tissues and cells may not be able to handle a sustained nutrient overload which might result in adverse effects [63]. Increased levels of circulating NEFAs are associated with increased systemic inflammatory conditions in humans [63]. Excessive fat mobilization due to a severe energy deficit impairs the cow’s immune function and fertility and leads to metabolic stress [15]. Therefore, altered lipid metabolism, increased concentrations of non-esterified fatty acids, excessive adipose stores and oxidative stress during the onset of lactation in dairy cattle, may negatively affect the inflammatory response of the individual to several pro-inflammatory diseases including metritis, mastitis and ketosis. This might explain the underlying biological pathways and interactions of the genes *DGAT1*, *LEP* and *TNF* and their associations with subclinical ketosis.

Interestingly, in this study the GWAS identified two highly significant SNPs (BovineHD4100010548: rs110651119; BovineHD1400000400: rs109221516, FDR < 1%) at around 25 Mb on BTA14 located in the gene lymphocyte antigen 6 family member H (*LY6H*), and two significant SNPs (BovineHD1400000443: rs133518905; BovineHD1400000446: rs136122630 at FDR < 1% and < 5%, respectively) at around 27 Mb located in the gene lymphocyte antigen 6 family member K (*LY6K*) (Table 2). The putative role of these two genes (*LY6H* and *LY6K*) in immune response has been reported in several previous investigations [65–67] and *LY6K* gene was reported to be a significant candidate gene for mastitis susceptibility in US and Chinese Holstein cattle [47, 68]. Therefore, these regions, underlying genes and biological pathway might be also associated with subclinical ketosis in later lactations.

Two other highly significant peaks for SCK2 were located on BTA6 (spanning around 78.7–98.6 Mb) and BTA20 (55–63 Mb) (Fig. 1b, Additional file 1). Three SNPs on BTA20 (at around 53 and 57 Mb) and a region on BTA6 (spanning around 88~93 Mb) were found to overlap between SCK1 and SCK2. One significant intronic SNP (BovineHD4100005290: rs110899052, FDR < 5%) on BTA6 at 87 Mb in this study was located in the gene casein alpha s1 (*CSN1S1*). Associations of two significant SNPs within the promotor region of the *CSN1S1* gene with milk yield traits in German Angeln dairy cattle were reported in a quantitative trait loci (QTL) mapping study performed by Sanders et al. (2006) [69]. The presence of the significant genomic regions near the casein gene cluster (at 87 Mb) affecting milk production and milk component traits has been reported in several breeds including North American, Dutch, and US Holstein cattle for milk fat, milk protein yield and protein deviations [53, 70], in German Holstein [71] and Brazilian Holstein cattle [72] for milk yield and fat yield; however, this region was not reported to be associated with ketosis in previous studies. This region and its related gene might be associated with the high fat:protein ratio and decrease in milk protein percentage in early lactation [73].

Four SNPs in the current study (BovineHD1100004827: rs110571223; BovineHD1100004831: rs43672311) within gene *BIRC6* and (BovineHD1100004868: rs110243275; BovineHD1100004913: rs42050566) within the gene *TTC27* at 15~15.3 Mb were significant (FDR < 5%) (Additional file 1) on BTA11. This result is in agreement with the result of a GWAS study on ketosis susceptibility in Jersey dairy cows, in which significant regions were reported on BTA11 between 14.9 to 16.9 [29]. This region was reported to be associated with genes and pathways that regulate several functions, including metabolism, mastitis, steroid hormone metabolism and innate immune system receptors [74, 75]. Several SNPs on BTA25, spanning around 25.1–27.9 Mb for SCK2 (Additional file 1) were also identified. This result is supported by a gene-based mapping analysis of metabolic adaptation and metabolic disease in dairy cattle, in which two genes (ENSBTAG00000031551: *PRSS53*; ENSBTAG00000000781: *HIP1*) at around 27 Mb on BTA25 were shown to be associated with blood BHB concentrations and metabolic disorders [1]. Moreover, one highly significant SNP identified in the current study (BovineHD2500007432: rs42072596, FDR < 1%) on BTA25 was previously reported to be associated with carnitine and glycerophosphocholine in milk of Danish Holstein cattle [76]. This SNP is an intron variant located within the gene *CLN3* which encodes a protein involved in lysosomal function (Gene ID: 1201). Mutations in this gene are associated with a neurodegenerative disease in humans which leads to reduced levels of carnitine in plasma [77]. Carnitine has been an important component of mitochondria involved in fatty acid β-oxidation and lipid metabolism [78]. Additionally, Buitenhuis et al. [31] reported a high correlation between milk carnitine and choline (0.86), and choline and BHB in milk (0.97). Moreover, Klein et al. (2011) showed that high values of glycerophosphocholine in milk throughout the lactation period are connected with a low ketosis incidence in dairy cattle [79]. Therefore, this significant SNP and its assigned gene (*CLN3*) might have an important biological function associated with subclinical ketosis and other correlated metabolic traits in dairy cattle. Another highly significant SNP (BovineHD2500007435: rs42071217, FDR < 1%) at around 26 Mb on BTA25 within the candidate gene *APOBR* was also identified in the current study, which was previously reported to carry a causative variant highly associated with milk levels of glycerophosphocholine in German Holstein dairy cattle [15]. Other putative functionally important candidate genes for SCK2 on chromosome 25 found in this study were *IL4R* (BovineHD2500007120: rs42062457), and *PSPH* (BovineHD2500007818: rs109700166) genes.

The significant peak found on BTA20 for SCK2 was located at 55.3~63.9 Mb. Several significant SNPs (FDR < 1%) on this region (32 SNPs) were located within or very close to the gene trio Rho guanine nucleotide exchange factor (*TRIO*) (Table 2). This gene encodes a large protein that functions as a GDP to GTP exchange factor (Gene ID: 7204) and was reported to be a significant candidate gene for maternal effect on weaning weight and milk yield traits in Blonde d’Aquitaine beef cattle [80]. It has also been shown that the *TRIO* gene controls leukocyte trans-endothelial migration during inflammatory conditions and other diseases, such as rheumatoid arthritis [81]. Thus, this significant region on chromosome 20 and its associated gene might be also related to the inflammatory response in transition dairy cow and the occurrence of metabolic disorders. Other positional candidate genes that were identified in this study on BTA20 (55–63 Mb) were *ANKH*, *MYO10* and *DNAH5*. This region (at 55–63 Mb) on BTA20, was not reported to be associated with ketosis or subclinical ketosis in previous studies, therefore this region seems to be a novel region for subclinical ketosis.

Of the 369 SNPs that were used as candidate SNPs (FDR < 5%) in SNP2GO for enrichment analysis, a total of 114 SNPs were overrepresented in 91 enriched GO terms (Additional file 2). Some of these terms showed a clear association with SCK2 including GO:0030073 (insulin secretion); GO:0001678 (cellular glucose homeostasis) and GO:0046883 (regulation of hormone secretion). In order to have a better insight of the key biological pathways and their association with subclinical ketosis, GO terms were summarized and visualized using REVIGO web-server (Fig. 5). Blue and green bubbles show the GO terms with more significant *P*-values. The size of the bubbles indicates the frequency of the GO terms. One important GO term in the scatterplot is a term associated with regulation of Rho protein signal transduction; many researches over the last decade have uncovered the molecular links between the RhoGTPase and the NF_*K*_B pathways, and its association with inflammation and immune response [82].

### Overlapping regions among SCK1 and SCK2

The only overlapping peaks between the SCK1 and SCK2 traits were in the regions identified on chromosome 6 at 88–93 Mb and chromosome 20 at 57 Mb. These regions affect milk BHB concentrations (subclinical ketosis) both in first and later lactations in cows. In dairy cattle milk production increases in later parities [83]. Production of more milk might be associated with increased milk fat content due to adipose mobilization and a higher fat:protein ratio [84].

## Conclusions

The genome-wide association analysis on MIR predicted BHB milk in this study identified several significant regions associated with subclinical ketosis in first (SCK1) and later (SCK2) lactations in North American Holstein dairy cattle. The significant regions were mostly located on BTA6, 14 and 20. Regions on BTA6 and BTA20 were found to overlap between SCK1 and SCK2. Several of the identified regions were reported in previous investigations as associated with ketosis biomarkers, milk production, clinical mastitis and immunity in dairy cattle. However, a novel region on BTA20 at 55–63 Mb was also identified, which was not previously reported. Functional analysis identified pathways and biological terms and processes involved in lipid metabolism and immune function. The results of this study can be used for further genetic analysis to identify genes and causal variants that affect ketosis and other correlated metabolic diseases.

## Methods

### Animals and data

The Canadian Dairy Network (CDN, Guelph, Ontario, Canada) provided pedigree, genotypes and estimated breeding values (EBV) of cows and proven bulls for MIR predicted milk BHB in first lactation (SCK1) and later lactations (SCK2). Milk BHB measurements have been collected as a part of routine phenotyping via MIR spectroscopy by Canadian DHI since January 2013 using an existing FOSS calibration model and MilkoScan FT+ (FOSS Analytical A/S, Hillerød, Denmark) [85]. The correlation between chemical methods for measuring milk BHB and predictions using MIR spectroscopy has been shown to be approximately 0.80 [27].

Genotypes of 24,657 Canadian Holstein bulls (9,856) and cows (14,801) with the BovineSNP50K (50 K, 41,097 SNPs) BeadChip (Illumina, San Diego, CA) were provided by CDN. The SNPs in the 50 K panel passed standard quality control measures used by CDN. These genotypes were then imputed to the high density (HD, 311,725 SNPs, after editing 777 K panel for redundant SNPs [86]) genotypes (with a reference population of 2,507 animals) using FImpute V2.2 software [87]. Quality control was performed on the imputed HD genotyping data using the snp1101 software [88] and SNPs with low call rate < 0.9, low minor allele frequency (MAF) < 0.01 and excess of heterozygosity > 0.15 were excluded. After quality control, a total of 298,210 SNPs remained which were used for further genome-wide association analysis.

### Estimation of breeding values for milk BHB

Estimated breeding values were calculated by CDN with a multiple-trait (9 traits) linear animal model [89] including metabolic disease traits, milk recording indicators, and body condition score. Milk BHB, or sub-clinical ketosis, for first and later (up to fifth) lactations is recorded at the first test-day from 5 to 45 days in milk (DIM), expressed as milk BHB in mmol/L and log transformed. Data for lactations greater than 2 are treated as repeated observations. The model equation for milk BHB at first lactation included the fixed effects of herd, year-season, DIM, age-season-parity, and random herd-year and animal additive genetic effects. For later lactations, a random permanent environment effect was added to account for repeated measures. Specific model details and assumptions can be found in Jamrozik et al. (2016).

### Calculating de-regressed EBV

The EBV for MIR predicted milk BHB were de-regressed as explained in Garrick et al. [90] to be used in the genome-wide association study (GWAS). The Garrick et al. (2009) regression procedure adjusts for ancestral information and eliminates shrinkage contained in the EBV; therefore, the de-regressed EBV only contains their own and the descendant’s information on each animal [90].

### Genome wide association study

The association analysis was performed using a single SNP regression mixed linear model implemented in the snp1101 software [91]. The mixed linear model used in this study was as below: *Y*_*i*_ = *μ* + *βg*_*i*_ + *a*_*i*_ + *e*_*i*._

where ***Y***_***i***_ is pseudo phenotype of the ***i***^***th***^ bull (bull’s de-regressed EBV); ***μ*** is the overall mean; ***β*** is the linear regression coefficient (allele substitution effect) of the SNP; ***g***_***i***_ is the SNP genotype of the ***i***^***th***^ bull (coded as 0 for BB, 1 for AB and 2 for AA SNP genotypes); ***a***_***i***_ is the random additive polygenic effect of the ***i***^***th***^ bull and ***e***_***i***_ is the random residual effect.

Assumptions of the model include ***a~N***(**0**,  ***Gσ a***^**2**^ ) in which **G** is the genomic relationship matrix [91] and ***σ a***^**2**^ is the polygenic additive genetic variance; **e** ***~N***(**0**, ***Rσ e***^**2**^) in which ***σ e***^**2**^ is the residual variance, where ***a*** and ***e*** are vectors of additive polygenic and residual effects, respectively. **R** is a diagonal matrix containing weights for the residual variance based on the reliabilities of the de-regressed bull EBV [91].

In order to account for multiple testing, genome-wise false discovery rate (FDR) of 5 and 1% were used to identify significant and highly significant associations, respectively. The inflation factor ***λ*** [91] and quantile-quantile (Q-Q) plots were also calculated to compare observed distributions of –log(P-value) to the expected distribution under the no association model for each trait.

### Enrichment analysis

Significant SNPs at the level of 5% FDR were mapped to the corresponding genes in Ensemble database (Ensembl 90, *Bos taurus* UMD3.1, http://useast.ensembl.org/Bos_taurus/Info/Index) using the getBM() function in R-biomaRt package (https://www.bioconductor.org/) [92]. Genes within ±100 kb from the identified significant SNPs were used for further functional analysis.

Functional analysis was performed to identify the biological pathways and gene-networks associated with the list of positional candidate genes using the Ingenuity Pathway Analysis software (IPA; Ingenuity System Inc., USA). Additionally, a candidate SNP enrichment analysis was performed to identify genomic annotations and associated Gene Ontology terms (GO terms) for each trait using SNP2GO R package, as explained by Szkiba et al. (2014). For the GO term enrichment analysis, the Ensembl version 90 genomic annotation file (for *Bos taurus* UMD 3.1 assembly) in conjugation with Ensembl gene ID file (from Ensembl version 91 with gene ID and GO term accession) was used. The SNP2GO “extension” and the “runs” parameters were set to 50 nucleotides and 100,000, respectively and a false discovery rate (FDR) of 1% was used to correct for multiple testing [93]. The collection of enriched GO terms (FDR < 0.01) resulted from the SNP2GO analysis were then summarized and visualized using REVIGO web server (http://revigo.irb.hr) [94, 95]. This analysis assists the interpretation of the result by reducing the number of redundant enriched GO terms, using a simple clustering algorithm, and produces a scatterplot, relying on semantic similarities [95]. For this analysis, the GO terms enriched for both traits and the P-values from SNP2GO were uploaded to REVIGO. Settings used for REVIGO program were: database: *Bos taurus*, semantic similarity: 0.5 (small), semantic similarity measure: SimRel.

## Additional files

Additional file 1: Excel file describing highly significant SNPs (genome-wise FDR < 5%) resulting from GWAS analysis using single SNP regression mixed linear model for subclinical ketosis in first lactation (SCK1) and subclinical ketosis in later lactations (SCK2) (XLS 91 kb)

Additional file 2: Excel file describing gene ontology biological process terms (GO terms) enriched among GWAS results using SNP2GO enrichment analysis for subclinical ketosis in first lactation (SCK1) and subclinical ketosis in later lactations (SCK2) (XLSX 24 kb)

## Abbreviations

BHB: β-hydroxybutyric acid
BHBA: β-hydroxybutyric acid
BTA: *Bos taurus* autosome
CDN: Canadian dairy network
FDR: false-discovery rate
GO: gene ontology
GWAS: genome-wide association study
IPA: ingenuity pathway analysis
MIR: Mid-infrared spectroscopy
NEFA: non-esterified fatty acid
Q-Q plots: Quantile-Quantile plots
QTL: quantitative trait loci
SCK1: subclinical ketosis in first lactation
SCK2: subclinical ketosis in second lactation and after
SNP: single nucleotide polymorphism
